# Inspiratory Muscle Training Included in Therapeutic and Training Regimens for Middle-Distance Runners

**DOI:** 10.3390/jcm14093180

**Published:** 2025-05-04

**Authors:** Paulina Okrzymowska, Krzysztof Mackala, Wojciech Kucharski, Krystyna Rozek-Piechura

**Affiliations:** 1 Department of Physiotherapy in Internal Medicine and Oncology, University of Health and Sport Sciences, 51-612 Wroclaw, Poland; paulina.okrzymowska@awf.wroc.pl; 2 Department of Track and Field, University of Health and Sport Sciences, 51-612 Wroclaw, Poland; krzysztof.mackala@awf.wroc.pl; 3 Department of Human Biology and Cosmetology, University of Health and Sport Sciences, 51-612 Wroclaw, Poland; wojciech.kucharski@awf.wroc.pl

**Keywords:** PowerBreathe, Threshold, physical performance, runners, physical activity

## Abstract

**Objectives**: The aim of this study was to evaluate the effectiveness of respiratory muscle training in runners in relation to gender and trainers (PowerBreathe and Threshold). **Methods**: This study comprised 32 athletes training in middle-distance running at a high sports level. The subjects were divided into groups depending on the applied breathing training (IMT): group IMT on the PowerBreath, group IMT on the Threshold, and the control group labeled sham-IMT. The following tests were performed on each athlete: spirometry, maximal inspiratory pressure, expiratory pressure, and physical performance. **Results**: A significant increase in the levels of the parameters VO_2_/kg, PEF, PI_max_, and PE_max_, as well as a decrease in lactic acid levels and an increase in lactate threshold in both sexes, were observed as a result of the training on the PowerBreathe device. There were no significant differences in the levels of the parameters VO_2_/kg, PEF, PI_max_, lactic acid, and lactate threshold in either sex after Threshold training. A significant increase in PE_max_ was found in the Threshold device training group. **Conclusions**: Most of the assessed parameters of physical fitness and lung ventilation function, along with the respiratory muscle strength of women and men running middle distances, increased significantly after the use of IMT on PowerBreathe, and these results were maintained in the third stud, in contrast to the use of IMT on Threshold, with which there was no significant improvement.

## 1. Introduction

The respiratory system can be a limiting factor in the physical performance of highly competitive athletes [1]. Respiratory muscle fatigue can interfere with athletes’ maximal performance by impairing blood flow and motor muscle perfusion [2]. Respiratory muscle fatigue is observed in athletes in long-term moderate-intensity and short-term high-intensity efforts [3,4].

During high-intensity exercise, the respiratory muscles absorb about 10% to 15% of total VO_2_ and become susceptible to fatigue [5]. In healthy individuals, the inability to maintain a high level of ventilation is considered a limiting factor for maximal aerobic capacity [6]. Hence, it is likely that respiratory muscle fatigue can significantly reduce the capacity of athletes [7]. Such exhaustion results in a metabolic reflex in which metabolites (i.e., hydrogen ions) accumulate in the respiratory muscles, which indirectly leads to reduced blood flow in the limbs during exercise, leading to decreased exercise tolerance and increased dyspnea, and thus reduced performance [7].

An essential sport in which inspiratory muscle training finds its application is running, especially when it is a sport that is readily used as a form of popular and accessible physical activity. In running, cardiorespiratory endurance and respiratory muscle strength are important and should be developed to improve health. When individuals perform high-intensity exercise or experience exertion, respiratory muscle fatigue reflexively increases the sympathetic activity and vasoconstriction of the exercising limb, resulting in blood flow being unable to reach the limb muscles [8,9,10]. Therefore, insufficient blood flow in the limbs decreases the oxygen exchange rate and increases feelings of pain and discomfort, thus affecting sports performance. Training is crucial to optimize exercise performance to achieve optimal strength and power and to prevent musculoskeletal injuries [11].

Johnson et al. (1996) suggested that reduced blood flow to the respiratory system during exercise can cause respiratory muscle fatigue [12]. Reduced available blood flow to the respiratory muscles can result in reduced oxygen content in the muscle cells and accumulation of metabolic by-products such as lactic acid. Lactic acid accumulation is associated with both an increased sensation of dyspnea and a reduced ability of the respiratory muscles to generate force [12].

The selection of appropriate training intensity is paramount to the efficacy of IMT. Research indicates that training intensity should be tailored to training goals. In the available literature, there are different variations in IMT depending on the training intensity. Intensity at levels up to 80% of maximum inspiratory pressure (MIP) lead to significant gains in inspiratory muscle strength, vital capacity (VC), and capacity and power output. Intensity at 60% of MIP improves fitness and power output, but to a lesser extent than 80% of MIP [6]. Studies also confirm the effectiveness of inspiratory muscle training with an initial load of 50% of MIP, with progressive increases in intensity, which improves inspiratory muscle strength and physical performance [13]. Increases in training intensity are also an important aspect of IMT programming. It allows the inspiratory muscles to adapt and avoid overload. Progression should be tailored to individual capabilities and training goals [14]. Regardless of the level of loading during IMT, the diaphragm, external intercostal muscles, and additional inspiratory muscles (including the sternocleidomastoid muscles, scalene muscles, and pectoral muscles) are engaged [15].

The available literature confirms that inspiratory muscle training improves the performance of athletes in some sports, such as cycling, running, and rowing, and can lead to changes in the functional parameters of the respiratory system [16]. Based on a review of 20 publications and 448 participants, the meta-analysis showed a significant positive effect of expiratory muscle training on sports performance in time trials, exercise endurance time, and repetitions in Yo-Yo tests. Inspiratory muscle strength and endurance improved in the majority of studies, which partly depended on the type of RMT used. Determining the kind of athlete most likely to benefit from RMT was limited by small sample sizes, different RMT protocols, and differences in outcome measures across studies. In summary, RMT can improve athletic performance [17,18].

Rożek-Piechura et al. evaluated the effectiveness of inspiratory muscle training on the lung function and physiological adaptation of long-distance runners during sports training at different intensities [19]. They divided the runners into three groups according to training intensity (IMT: PowerBreathe trainers, Threshold, and control group) and found that using higher-intensity IMT significantly improved all lung ventilation parameters. In addition, lactate accumulation was significantly reduced after training. Physiological characteristics (VO_2max_/kg) and respiratory muscle strength variables improved significantly in the highest-intensity group (PowerBreathe) after 8 weeks of training [19].

Middle-distance running includes runs at 800 m and 1500 m. These include both aerobic and anaerobic exercise. A study of 1500 m runners found that training the inspiratory muscles can increase respiratory muscle strength and improve athletic performance [20]. In contrast, the 800 m distance in exercise physiology is referred to more as anaerobic exercise [21,22]. It is puzzling whether the effect of training the respiratory muscles will be the same in runners competing at 800 m [23]. Few studies have investigated the impact of this training on short-distance sports such as 800 m running. Ohya et al. (2016) found that shorter running exercises can induce inspiratory muscle fatigue [24]. Therefore, there is a need to better document whether training the inspiratory muscles is helpful for people performing middle-distance runs, such as 800 m runs [24].

Physiologically, the effect of inspiratory muscle training is to increase the strength of the higher thoracic and neck muscles, improving thoracic geometry due to an increase in VC. Following training, increased diaphragm thickness also improves lung ventilation [25]. Respiratory muscle training can prolong fatigue, improving athletic performance, arterial blood, gas, and acid–base balance [26]. In addition, it increases pulmonary oxygen uptake and reduces blood lactate concentration, VO_2_, cardiac minute volume, and inspiratory muscle fatigue under normoxic and hypoxic conditions [27].

Numerous physiological and demographic factors, and the absence or presence of disease, influence lung function. These include body size, gender, age, and ethnicity. In general, larger individuals tend to have better lung function, whereas women typically exhibit lower lung function values [28].

The available literature indicates the presence of three-dimensional sexual dimorphism in lung morphology, as well as kinematic differences in breathing between men and women. Men additionally show greater changes in size and shape during respiration. It is presumed that this is likely due to different respiratory muscle engagement, which translates into a more pyramidal lung shape in men and a more prismatic lung shape in women. Interestingly, women are less prone to diaphragm fatigue compared to men, despite the presence of greater ventilatory constraints and more respiratory work during exercise. The unequivocal explanation for this mechanism of diaphragm fatigue resistance in women is not unknown, but it is speculated that it may be related to gender differences in respiratory muscle recruitment [29].

Available electromyographic findings indicate that during intense exercise, women have higher activation of accessory inspiratory muscles, such as the sternocleidomastoid muscle and the scalene muscles, compared to men. Interestingly, diaphragm activation (EMGdi) did not differ between the sexes, which may suggest that women may use the activation of accessory inspiratory muscles as a strategy to minimize diaphragm fatigue [30]. According to the literature, there is a difference in physiological responses between men and women when overcoming inspiratory resistance. Women show a higher resistance to diaphragm fatigue during inspiratory resistance exercise. Research confirms that the time to exhaustion (the occurrence of fatigue symptoms) was significantly longer in women than in men, despite a similar degree of diaphragm strength reduction at comparable fatigue levels [31]. Existing gender differences in respiratory system structure and function may affect the response to inspiratory muscle training. Women, despite having smaller lung volumes and higher work in breathing, show greater resistance to diaphragm fatigue, which may suggest differences in adaptive mechanisms. Although IMT leads to similar increases in inspiratory force in both sexes, the effects on physical performance may be more variable and dependent on individual conditions and environmental conditions [32]. Consequently, conducting further research on the physiological effects of inspiratory muscle training according to the sex of the subjects is necessary to determine more precisely what the adaptation to inspiratory muscle training is and how this information can be used in clinical and sports practice in men and women.

Accordingly, the present study aimed to verify whether the addition of inspiratory muscle training at different intensities (PowerBreathe and Threshold) to traditional training in middle-distance runners during the preparation period increases the physiological effect of the athlete’s preparation for competitions and whether this is related to the gender of the subjects studied.

## 2. Materials and Methods

### 2.1. Participants

Thirty-two athletes, all middle-distance runners, took part in this study. The inclusion criteria were age 19–26, at least 5 years of training experience, and an elite level of sport. The exclusion criteria were the presence of bronchial asthma or any other respiratory disease requiring medication, a history of spontaneous pneumothorax (collapse of the lung due to trauma such as rib fracture), and status post eardrum rupture.

Sample size estimation was carried out using G*Power software version 3.1 [33]. Based on the adopted study design and three repeated measurements, the minimum required sample size was 30 participants for an effect size of 0.20, an alpha level of 0.05, and a power of 0.95.

The subjects were randomly divided according to a distribution table into six groups according to gender and type of IMT used. The subjects were divided into the following subgroups:

– Five female runners who received additional IMT with the PowerBreathe device;
– Six male runners who received additional IMT with the PowerBreathe device;
– Five female runners who received additional IMT with the Threshold IMT device;
– Six male runners who received additional IMT with the Threshold IMT device;
– The control group (sham-IMT), consisting of five female runners who received additional IMT using the Threshold IMT device with a 5% load;
– The control group (sham-IMT), consisting of 5 male runners who received additional IMT using the Threshold IMT device with a 5% load.

Table 1 shows detailed characteristics of the subjects. All groups were homogeneous, and there were no significant differences in anthropometric variables between the groups evaluated. All runners completed the same general preparatory training cycle.

### 2.2. Training Characteristics of Middle-Distance Runners

Training for middle-distance, i.e., 800–1500 m, differs significantly from training for long-distance races: 5 km, 10 km, and especially from a half marathon or a marathon. These differences mainly result from the volume of endurance training. A middle-distance runner performs a much smaller volume of training focused on aerobic endurance (65–79% max HR or 1–2 mmol LA) as a foundation for special endurance. The running training volume varies depending on the training period: general preparation, special preparation, BPS, or the competition period. It also depends mainly on the training goal for a given year and, above all, on the sports level of the runner. The quality of running training also changes depending on the training period, i.e., the intensity and form of its execution. During the special preparation period, the running training volume decreases and its intensity increases. In middle-distance running, in contrast to long-distance running and marathons, there is a high concentration of lactic acid SP (special endurance: 85–95% max HR or 6–8 mmol LA), and muscle work shows a strong anaerobic character (Tempo interval training: 85–95% max HR or 8–10 mmol LA). A runner specializing in middle-distance running, in addition to working on endurance, realized in various forms, must concentrate on working on strength, both with external resistance and running strength. Much of the training is intended for speed training and general fitness, expressed by work on mobility, technique, general strength, and medicine balls.

The above training description refers to the Polish school of middle-distance running, whose athletes have achieved many international successes in the last decade. It is a matrix for preparation training with a strong focus on an individual approach to the load and its effects. These guidelines also fit into the global trends of middle-distance running training. The load indicator is the heart rate and lactate level, which are generally recorded and implemented individually by the players. Our training program, which the tested runners implemented, was prepared on these guidelines.

Table 2 shows the training of one of several weekly microcycles implemented during the tested runner’s preparatory period. Each training accent, e.g., GE1, general endurance in the first heart rate range (65–79% max HR), has an assigned number of implemented training units in this microcycle. The control of the implemented training (load) for our athletes was monitored daily by continuous heart rate measurement (POLAR H10, Polar Electro Oy, Kempele, Finland) and lactate (LA) level (Accutrend lactate BM).

### 2.3. Methods

This study was conducted during the preparation period for athletes in the introductory mesocycle and the general preparation mesocycle. These mesocycles aim to prepare and improve running endurance in the first and second intensity ranges of overall strength, rhythm, and fitness. The planned training of the respiratory muscles was carried out over eight weeks and started with performing a muscle strength assessment, which is essential for calculating the correct training load.

**Figure 1:**
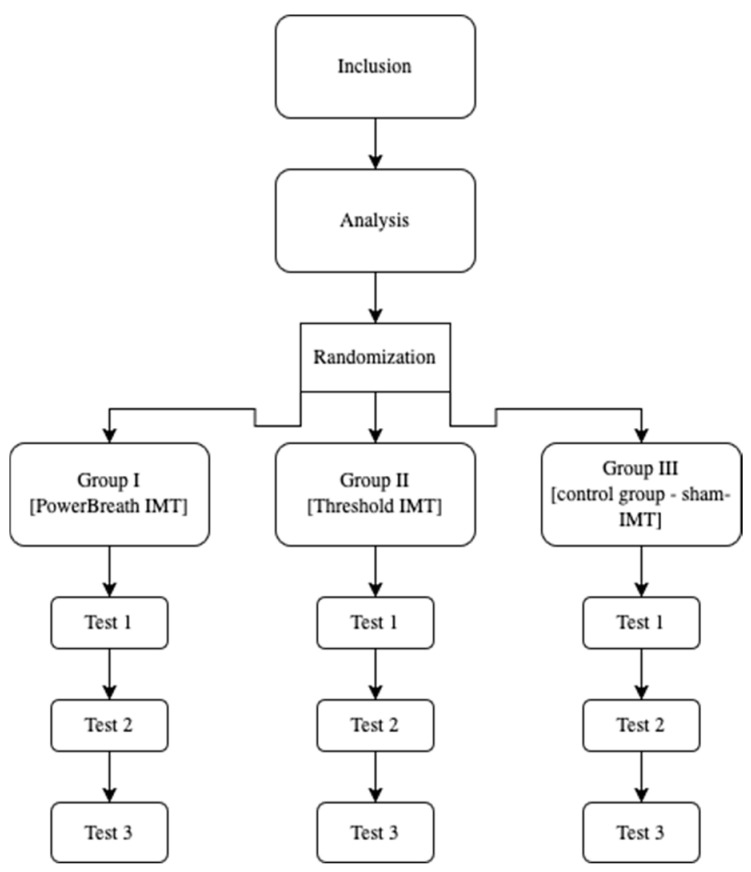
Design of this study.

The tests were performed three times: before the start of the preparatory training period with the simultaneous start of inspiratory training (test 1), a second test after eight weeks of inspiratory training (test 2), and a third test after a further six weeks (test 3), i.e., at the end of the 14-week preparatory period (Figure 1).

Spirometry was performed on each athlete using Master Screen Pneumo (CareFusion, Germany) equipment and assessed vital lung capacity (VC), forced expiratory volume in one second FEV_1_ [L/s], and peak expiratory flow PEF [L/s]. The absolute values measured during this study were expected [34] and were calculated hypothetically based on sex, age, height, and weight. A regression formula corresponding with the standards developed by the European Coal and Steel Community in Luxembourg was used to calculate the normal values [35]. Respiratory muscle strength was assessed by measuring the maximum inspiratory pressure PI_max_ and expiratory pressure PE_max_ expressed in units of kilopascals, which correspond to the measurement of the maximum pressure developing in the first second of inspiration and expiration/pressure developing during inspiration and expiration. The test was performed using a special attachment for the spirometer mentioned above. The unit conversion rate resulting from the difference between the units used in the training device and the units obtained during the PI_max_ and PE_max_ tests was used to calculate the inspiratory muscle training loads [36]. Physical capacity was assessed using the Vyntus CPX device. A maximal progressive treadmill test was used. The protocol started with a 5-min warm-up at 6 km/h. During the central part of the test, the speed was increased by 2 km/h every 3 min. Once the speed reached 16 km/h, the speed was held constant, but the treadmill’s incline increased by 2.5% every 3 min. Criteria for completing the test included voluntary exhaustion or reaching the participant’s maximum HR (calculated as 220 beats per minute-age).

The following parameters were measured during this study: maximal oxygen uptake VO_2max_ [mL/min], VO_2_/kg [(mL/min)/kg], maximal carbon dioxide output VCO_2max_, [mL/min], minute ventilation VE [L/min], and respiratory exchange rate RER = VCO_2_/VO_2_ [min]. To assess the extent of anaerobic metabolism during exercise, lactic acid level [m/mol] and lactate threshold [min] were measured using a portable analyzer (Lactate Scout+, Leipzig, Germany) from blood drawn from the fingertip.

### 2.4. Inspiratory Muscle Training Using the PowerBreathe Device

**Figure 2:**
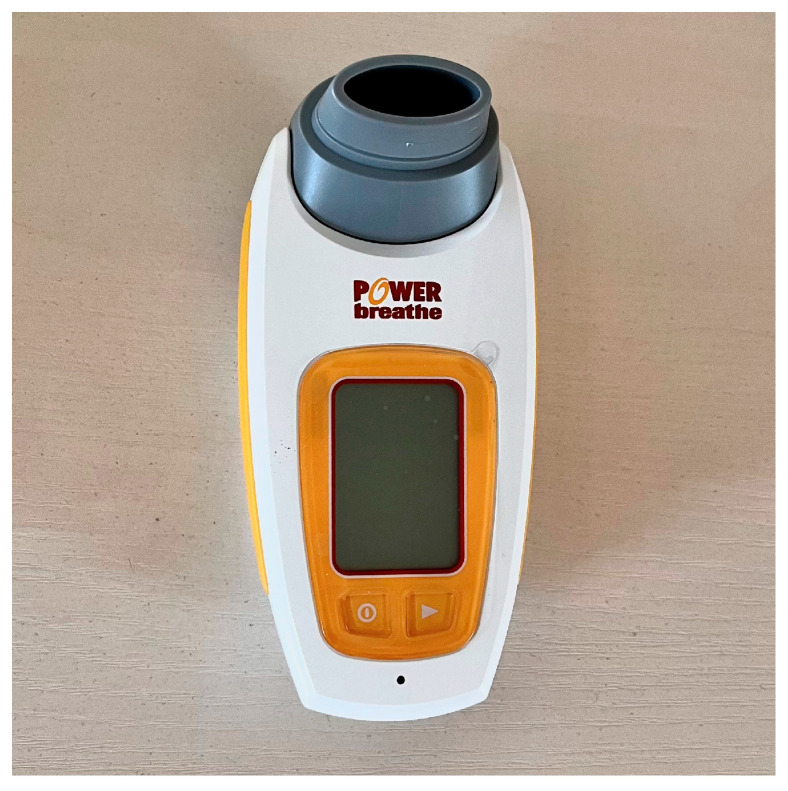
PowerBreathe device.

Inspiratory muscle training using the PowerBreathe device (Figure 2), providing sub-threshold loading (PowerBreathe K1; PowerBreathe International Ltd., England), was used in group PowerBreathe-IMT, in two sessions daily, five times a week. During IMT, the subjects performed 30 maximal inhalations, maintaining a diaphragmatic breathing pattern (Table 3) [10]. One session per week was conducted under supervision, and the remaining four sessions were performed at home. The athletes performed IMT twice a day (in the morning and evening). To ensure the effective execution of the training program, each participant was required to maintain a training dairy. Due to the scale of the PowerBreathe device, the obtained PI_max_ measurements (kPa) were converted to the device unit (H_2_O) as follows: 1 kPa = 10.2 cm H_2_O [36].

### 2.5. Inspiratory Muscle Training Using the Threshold Device

**Figure 3:**
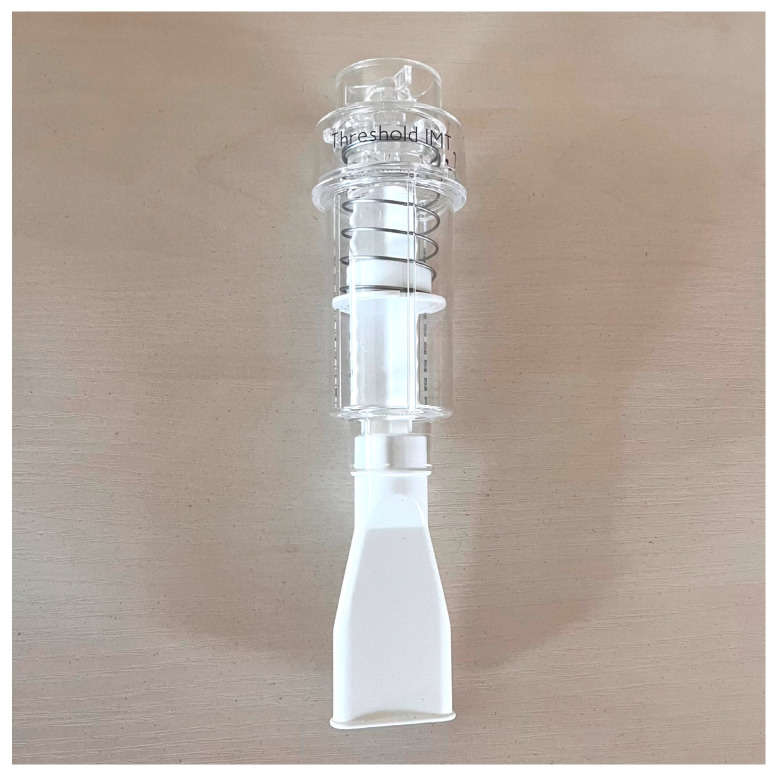
Threshold device.

The athletes in the group used the Threshold (Figure 3) device for eight weeks, in two sessions per day, five times weekly. This included performing 30 maximal inhalations and maintaining a diaphragmatic breathing pattern [37]. The IMT load in these groups started with a load of 30% PI_max_, which was increased each week to 50% PI_max_ (Table 4). The athletes performed IMT twice a day (in the morning and evening). To ensure the effective execution of the training program, each participant was required to maintain a training dairy. Due to the scale of the Threshold device, the obtained PI_max_ measurements (kPa) were converted to the device unit (H_2_O) as follows: 1 kPa = 10.2 cm H_2_O [38].

### 2.6. Inspiratory Muscle Training Sham-IMT

IMT in group 5, and group 6 (control), sham-IMT, included low-intensity inspiratory muscle training with a load of 15% PI_max_ in the Threshold device (Figure 3, Table 5). The athletes trained once a day in the morning. To ensure the effective execution of the training program, each participant was required to maintain a training dairy [39].

### 2.7. Statistical Analysis

Statistica version 7.0 software from StatSoft (Dell, Round Rock, TX, USA) was used to analyze the results. The Kolmogorov–Smirnov and Lilliefors tests were used to assess the normality of the distribution of variables. Basic descriptive statistics were calculated. Differences in spirometric, respiratory muscle, and physical fitness variables between groups and measurements were assessed using ANOVA with repeated measures and the post hoc NIR test. Differences were considered statistically significant at *p* < 0.05. The effect sizes for the magnitude of statistically significant group differences were calculated, and the effect sizes were expressed as small at 0.2, while 0.5 showed a moderate effect and 0.8 showed a strong effect.

## 3. Results

This section concisely and precisely describes the experimental results, their interpretation, and possible experimental conclusions. It starts by evaluating the runners’ physical performance parameters concerning gender and the measurement points conducted.

**Figure 4:**
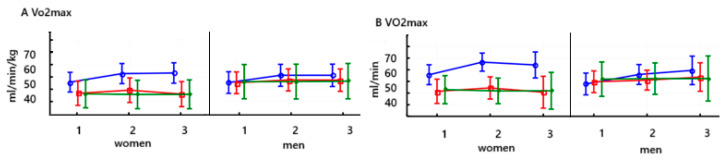
Means and standard deviations of the athletes’ maximal oxygen consumption (VO_2max_) (mL/min/kg (**A**) and mL/min (**B**)) according to gender and training: 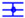 PowerBreathe IMT, 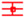 Threshold IMT, 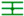 Sham IMT—control group, and trials 1, 2, 3, *p* = 0.123.

In women, VO_2_/kg demonstrated a highly significant increase after an 8-week training period on PowerBreathe (*p* < 0.000) and remained at the same level after the end of training for a further 6 weeks (Test 3). In men, VO_2_/kg increased significantly in the second study (*p* < 0.05). No significant changes were observed in women after training on the Threshold device, and the same trend was observed in men. After the training was applied, no significant differences were observed in the control group, both for women and men. The baseline values of this parameter were similar in men and women (Figure 4A and Table 6).

In women, the VO_2max_ (ml/min) value saw a highly significant increase after 8 weeks of training on the PowerBreathe trainer (*p* < 0.000) and maintained a higher value after training for another 6 weeks (test 3). In men, the VO_2max_ parameter value saw a highly significant increase in the second study after using the PowerBreathe trainer (*p* < 0.001) and maintained this value after training in the third study (*p* < 0.001). The women’s VO_2max_ value increased significantly after 8 weeks of training on the Threshold device (*p* < 0.05). It then decreased significantly (*p* < 0.01), indicating that the effect of this training was not maintained. Baseline VO_2max_ in men after 8-week training on the Threshold device increased significantly after its application (*p* < 0.05) and remained unchanged in the third study. No significant changes were noted in the control groups, both for men and women. In each group, women had a lower baseline VO_2max_ than men (*p* < 0.000) (Figure 4B and Table 6).

**Figure 5:**
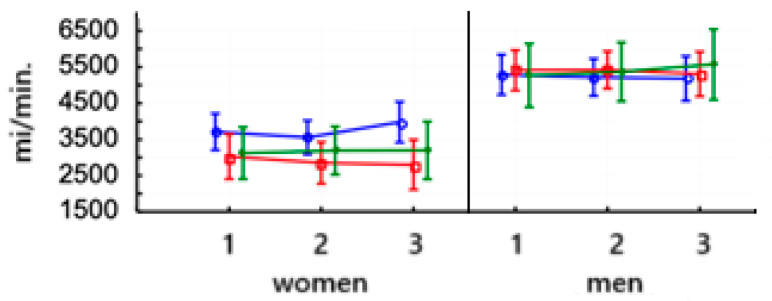
Means and standard deviations of the athletes’ carbon dioxide output (VCO_2max_—mL/min) according to gender and training: 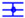 PowerBreathe IMT, 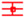 Threshold IMT, 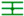 Sham IMT—control group, and trials 1, 2, and 3.

There was only a tendency for a decrease in VCO_2max_ for the women in both the Powerbreathe and Threshold training sessions (Figure 5, Table 6). In all studies, VCO_2max_ did not change significantly in men regardless of the use of inspiratory training. The baseline values of this parameter were markedly lower in women (*p* < 0.001).

**Figure 6:**
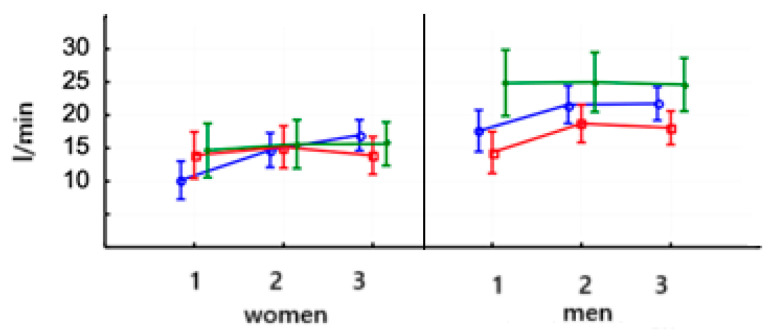
Means and standard deviations of the athletes’ respiratory exchange ratio (RER) according to gender and training: 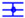 PowerBreathe IMT, 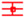 Threshold IMT, 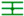 Sham IMT—control group, and trials 1, 2, and 3.

The RER value (Table 6) in women saw a highly significant increase after 8 weeks of PowerBreathe training (*p* < 0.000). In men, baseline RER increased considerably after 8 weeks of PowerBreathe training (*p* < 0.01) and was maintained at the value from the second study (Figure 6). All studies showed no significant differences in women after using the Threshold device. In men, after using Threshold training, the parameters saw a highly significant increase in the second study (*p* < 0.001) and remained at the same level in the third study. In the control group, the value of these parameters remained the same in both men and women. The baseline values of this parameter were markedly lower in women (*p* < 0.001).

**Figure 7:**
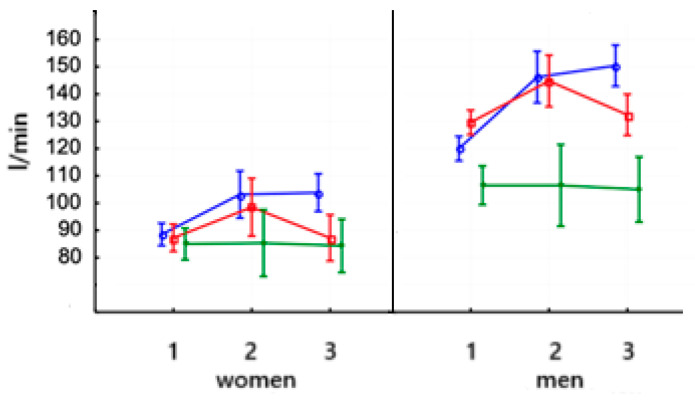
Arithmetic means and standard deviations of the athletes’ minute ventilation (VE—L/min) according to gender and training: 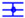 PowerBreathe IMT, 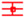 Threshold IMT, 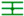 Sham IMT—control group, and trials 1, 2, and 3.

The value of VE in women and men saw a highly significant increase after 8 weeks of training on PowerBreathe and Threshold (*p* < 0.000) (Figure 7) (Table 6). After Threshold, the values of this parameter significantly decreased in the third study in both gender groups. In the control groups, women and men showed no significant changes regardless of the test. In each group, women had a lower baseline VE value than men (*p* < 0.000).

**Figure 8:**
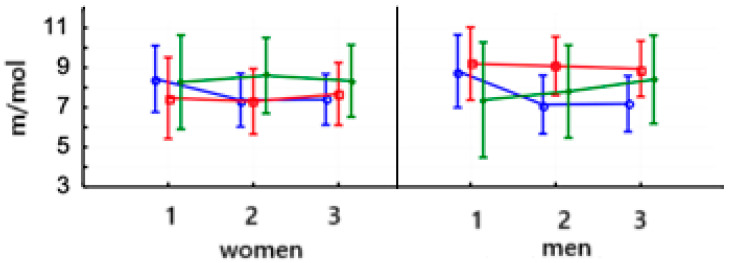
Arithmetic means and standard deviations of the lactic acid levels in athletes according to gender and group: 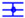 PowerBreathe IMT, 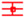 Threshold IMT, 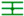 Sham IMT—control group, and trials 1, 2, and 3.

When we considered this, lactic acid levels in women after PowerBreathe training decreased significantly (*p* < 0.01) and remained at this level. The same trend was observed in men (*p* < 0.001) (Table 6). There were no significant differences in the values of this parameter in both women and men in the Threshold and control training groups (Figure 8). The baseline values of lactic acid levels were not significantly different.

**Figure 9:**
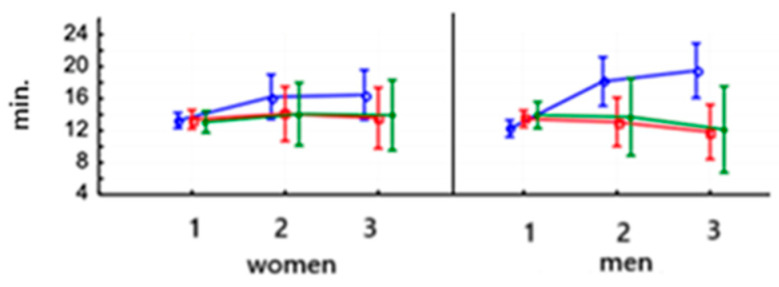
Means and standard deviations of the lactate threshold in athletes according to gender and training: 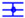 PowerBreathe IMT, 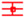 Threshold IMT, 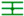 Sham IMT—control group, and trials 1, 2, and 3.

The value of lactate threshold in both women (*p* < 0.01) and men (*p* < 0.001) after PowerBreathe (Figure 9) training increased significantly in the second study and remained the same in the third study (Table 6). There were no significant differences in this parameter between women and men after training on the Threshold device or in the control group. The baseline values of this parameter were not significantly different in women and men.

The subsequent phase of the investigation entailed a comprehensive review of the runners’ spirometric parameters and respiratory muscle strength, with a particular focus on gender-related differences. The pulmonary ventilation abnormalities and decreased inspiratory muscle strength assessment showed no abnormalities compared to norms. The subjects did not have abnormal lung ventilation compared to the normative values, and no typical respiratory muscle strength abnormalities. The baseline values of respiratory muscle strength were within the physiological range, up to 80% of the normal value.

**Figure 10:**
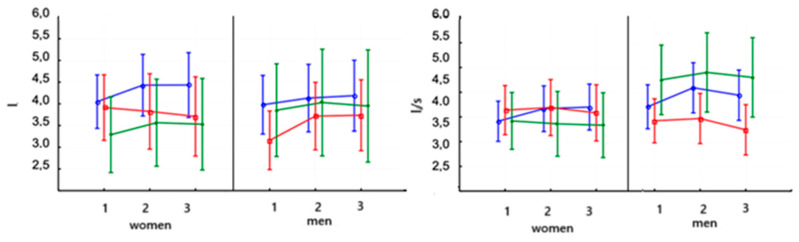
Means and standard deviations of the vital capacity (VC—L) and the forced expiratory in one second (FEV_1_—L/s) intensity capacity according to gender and training: 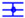 PowerBreathe IMT, 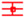 Threshold IMT, 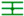 Sham IMT—control group, and trials 1, 2, and 3.

The VC value in women increased significantly after the 8-week training on PowerBreathe (*p* < 0.05) and was maintained at the same level after the training (Table 7). No significant differences were observed in men after PowerBreathe training (Figure 10, left). No significant changes were observed in women after training on the Threshold device. VC in men increased significantly after training on the Threshold device (*p* < 0.001) and remained at the same level after training. In the control group, both women and men showed no significant differences after the applied training. The baseline values of this parameter were at similar levels in women and men.

The value of FEV_1_ in women increased significantly (*p* < 0.001) after applying PowerBreathe training and remained at the same level in the third study (Figure 10, right, and Table 7). FEV1 increased significantly (*p* < 0.001) in men after PowerBreathe training. There were no significant differences in FEV_1_ parameter levels after Threshold training in women and men. In the control group, no significant differences in FEV_1_ parameter values were observed in either women or men. The baseline values of this parameter were not significantly different in either women or men.

**Figure 11:**
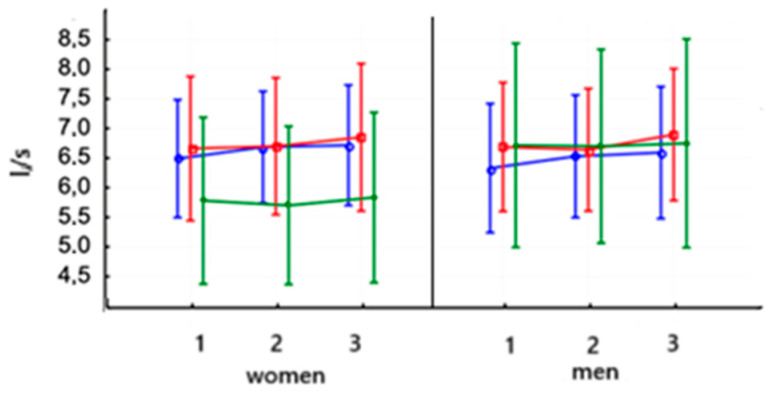
Means and standard deviations of athletes’ peak expiratory flow (PEF—L/s) according to gender and training: 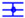 PowerBreathe IMT, 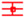 Threshold IMT, 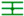 Sham IMT—control group, and trials 1, 2, and 3.

Figure 11 and Table 7 present the PEF parameter value in women and men after PowerBreathe training, which did not increase significantly in the second study. There were no significant differences after training on the Threshold device or in the control group in both women and men. The baseline values of this parameter were not significantly different in men and women.

**Figure 12:**
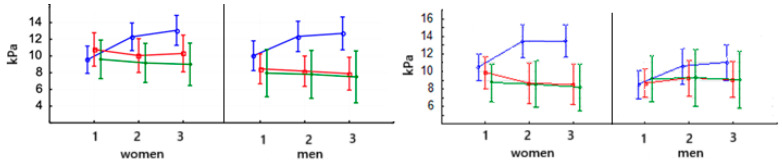
Means and standard deviations of the athletes’ inspiratory muscle strength (PI_max_ kPa, right side) and (PE_max_—kPa, left side) according to gender and training: 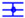 PowerBreathe IMT, 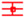 Threshold IMT, 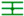 Sham IMT—control group, and trials 1, 2, and 3.

In women, after PowerBreathe training, the PI_max_ parameter value increased significantly in the second study (*p* < 0.000) (Table 7). The PI_max_ value in men increased significantly (*p* < 0.000) after PowerBreathe training and was maintained at the same level in the third examination. There were no significant differences in the values of this parameter after Threshold training in either men or women. The baseline values of this parameter were not significantly different in men and women (Figure 12).

Figure 12, right, presents the PE_max_ in women and men after PowerBreathe training. The value of this parameter in women and men increased significantly in the second study (*p* < 0.000) and remained at the same level in the third study (Table 7). After training on the Threshold device in women, the PE_max_ value significantly decreased in the second study (*p* < 0.05) and stayed at this level during the third study. There were no significant differences in the values of this parameter after Threshold training in men. There were no significant differences in PE_max_ values after training in the female and male control groups. The baseline values of this parameter were not significantly different in men and women.

## 4. Discussion

Our study showed a positive effect of training on the PowerBreathe device in both women and men, and on the Threshold device on VO_2max_. In women, the increase in VO_2max_ after training on the Threshold device was maintained in the third study. In contrast, there were no significant changes in VO_2max_ in the control group. The initial VO_2max_ value was significantly lower in women in all groups.

Our research also showed increased inspiratory muscle values (PI_max_) after training on the PowerBreathe machine and expiratory muscle values (PE_max_) in both women and men. A significant increase in FEV_1_ values was also observed in both women and men after PowerBreathe training. There was no significant change in FEV_1_ levels after Threshold training in women, while in men, the value was maintained in the second study and significantly decreased in the third study. On the other hand, peak expiratory flow only increased substantially in the third study in both sexes, six weeks after the PowerBreathe training.

The effects of respiratory training were studied by Williams et al., who evaluated the impact of training the respiratory muscles on the Threshold device. They examined seven long-distance runners—five men and two women. Before the procedure, they measured the following parameters in each participant: VO_2max_, PI_max_, breathing endurance time (BET) at 60% PI_max_, endurance run time (ERT) at 85% VO_2max_, HR, minute ventilation, VO_2_, ratings of perceived dyspnea (RPD), and blood lactate concentration. After 4 weeks of training, they measured the values of the above parameters again. They observed a significant increase in PI_max_ and BET, while no significant differences existed in the other parameters [40]. The self-reported results showed that the Threshold and PowerBreathe device training significantly improved these parameters. An increase in VO_2_ max in both men and women was observed after training on the PowerBreathe and Threshold devices, showing increased muscle strength. A significant result was that after six weeks, the high score was maintained only in the group after PowerBreathe training. However, the value increased in the men and was maintained in the second study.

Furthermore, it is worth noting that, in each group, the baseline VO_2max_ value was lower in women than in men. An explanation for obtaining such results is the use of 8 weeks of respiratory training in our study. Thus, this would indicate the validity of the length of respiratory training in training routines in runners.

Rożek-Piechura et al. (2020), on the other hand, evaluated the effectiveness of inspiratory muscle training (IMT) at different intensities (PowerBreathe, Threshold IMT, and control group) on lung function and physiological adaptations of long-distance runners undergoing sports training [19]. They assessed spirometric parameters, respiratory muscle strength (PI_max_, PE_max_), and spiroergometric parameters: heart rate, VO_2max_, VCO_2max_, maximum ventilation, and respiratory exchange ratio. The group that used the PowerBreathe device significantly increased all physiological and fitness variables assessed. In the group with IMT on the Threshold device, only ventilation VO_2max_, VE, and RER were increased considerably to levels similar to those observed in the PowerBreathe group. In the control group, only a significant reduction in saturation was observed. Higher-intensity IMT substantially improved all lung ventilation and respiratory muscle strength variables tested. Lactate levels were also significantly reduced after training. Physiological characteristics (VO_2max_/kg) and respiratory muscle strength variables improved significantly in the group that used the PowerBreathe device after 8 weeks of training [19]. The results of our study confirm these trends. In our research, the RER and VE parameter scores in both men and women increased after training on the PowerBreathe device. After Threshold training in men, there was an increase in the RER parameter. In contrast, the VE parameter value increased significantly in the second study, followed by a significant decrease in the third study.

Our study confirmed that women had lower VO_2max_ values than men, consistent with results from other authors [41,42]. The differences in VO_2max_ values are because women tend to have smaller hearts and lungs and lower hemoglobin mass than men, which may limit their ability to deliver oxygen to working muscles [42].

Our study showed improved inspiratory muscle strength in group I (PowerBreathe-IMT). The training effect obtained in the survey after using the PowerBreathe trainer, which is associated with improved physical performance in this group, may be due to enhanced musculo-vascular function [43]. Increased inspiratory muscle strength can inhibit the metabolic reflex during exercise, preventing the effect of sympathetic vasoconstriction in the locomotor muscles [44]. The women achieved lower minute ventilation (VE) than the male subjects. Other researchers also confirm this [45]. In women, the lower ability to generate increased ventilation during exercise is due to reduced airway diameter and lung volume. This difference results in lower peak expiratory flow and vital capacity and, consequently, lower minute ventilation values [46].

Different results were obtained by Cunha et al. (2019), who conducted a study on the effect of respiratory muscle training in professional swimmers, during which they measured inspiratory muscle strength, lung function, and perceived dyspnea [47]. They divided 32 participants into a test group that performed 30 inhalations twice a day, five times a week, for 12 weeks at a pressure threshold load equivalent to 50% of maximum inspiratory pressure, while the control group performed inhalations at the same frequency but at 15% load. After the experiment, they found no differences in swimming performance, inspiratory muscle strength, vital capacity, expiratory volume in the first second, peak expiratory flow, and perceived dyspnea between the groups after 12 weeks of intervention [47]. Such different results can most likely be attributed to the magnitude of the applied load on the respiratory muscles at 50%, given that it was applied to swimmers in whom sports training in the water already forces a higher load on these muscles. In our study, respiratory training began with a load of 50%, but the load was increased every fortnight to 70%. This confirms that the size of the training load can be of great importance in its selection for high-performance athletes.

On the other hand, Hartz et al. (2018) conducted a study on the effects of respiratory muscle training on respiratory muscle strength and resistance and handball athletes’ PP (physical performance) [48]. They studied twenty 20-year-old (±3) male athletes assigned to an experimental or placebo group. Using a cardiopulmonary exercise test, they assessed respiratory muscle strength by measuring maximal inspiratory and expiratory pressure (PI_max_ and PE_max_) and physical performance. After 12 weeks, they observed a significant difference in PI_max_ and PE_max_ values after training in the experimental group and PI_max_ in the placebo group [48]. These align with our results, in which a significant increase in PI_max_ parameter values was observed in the women and men after PowerBreathe training. There was no significant change in the level of this parameter in the female groups.

A limitation of this study was the size of the group. The group size is due to the inclusion criteria and the specificity of our population, which is related to the athletes’ high level of athletic training.

To summarize the results of our study, it should be stated that most of the assessed parameters of physical fitness and lung ventilation function, together with the respiratory muscle strength of female and male middle-distance runners, increased significantly after the application of IMT using PowerBreathe, and the results were maintained in the third study, in contrast to the use of IMT using Threshold, with which there was no significant improvement. Inspiratory muscle training can be successfully implemented as a complementary element during the preparation period in the general preparation phase of middle-distance runners.

## 5. Conclusions

The findings of the present study indicate that baseline values of parameters such as VO_2__max_, VCO_2__max_, and VE were lower in women across all analyzed groups. In contrast, parameters including VO_2_/kg, VC, FEV_1_, PEF, PI_max_, PE_max_, blood lactate concentration, and lactate threshold were comparable between male and female runners.

The incorporation of the PowerBreathe device training into the running regimen resulted in statistically significant adaptations in VO_2_max, VO_2_/kg, VE, RER, FEV_1_, PEF, PI_max_, and PE_max_ in both women and men. Simultaneously, a reduction in blood lactate concentration and an increase in lactate threshold were observed, indicating an improvement in metabolic efficiency.

In contrast, training with the Threshold device did not induce significant changes in VO_2_/kg, PEF, PI_max_, blood lactate levels, or lactate threshold in either sex. Moreover, the VCO_2__max_ parameter remained unchanged regardless of sex or the type of device used. Notably, an increase in RER was observed in men following Threshold training, while no significant change in this parameter was detected in women.

A particularly noteworthy finding was the increase in expiratory muscle strength (PE_max_) observed in both women and men after completing the PowerBreathe training program. Importantly, this adaptive effect persisted at the same level six weeks after the cessation of training. In contrast, following the Threshold training program, a decrease in expiratory muscle strength was noted in women, while no significant changes were observed in men.

Based on the obtained results, it can be concluded that high-intensity inspiratory muscle training using the PowerBreathe device is more effective than training with the Threshold device. It demonstrates superior efficacy in enhancing most spirometric parameters, respiratory muscle strength, and overall physical performance in both women and men.

## Figures and Tables

**Table 1:** Means, values, and standard deviations of age, weight, height, and BMI of athletes according to gender and type of training.

Variable	Sex	Group I [PowerBreathe IMT]	Group II [Threshold IMT]	Group III [Control Group]
Age [years]	W	23.3 ± 1.83	22.0 ± 3.4	21.5 ± 2.82
	M	22.3 ± 2.98	23.5 ± 3.54	22.5 ± 1.9
Height [cm]	W	1.63 ± 0.04	1.69 ± 0.06	1.61 ± 0.08
	M	1.82 ± 0.06	1.82 ± 0.04	1.84 ± 0.03
Body weight [kg]	W	55.5 ± 3.24	62.0 ± 1.65	51.0 ± 8.38
	M	70.4 ± 5.46	75.2 ± 4.32	76.5 ± 3.56
BMI [kg/m^2^]	W	21.43 ± 0.4	22.03 ± 0.33	20.15 ± 0.72
	M	22.39 ± 0.84	22.65 ± 1.3	22.71 ± 0.43

**Table 2:** A middle-distance training program is included in each weekly microcycle during the general preparation phase.

Type of Training Modality (Workout)	Training Session (7-Day Microcycle) Middle Distance	
	Number of Performance (n)	Mileage (km)
Strength endurance (combined with circuit training)	1	
GE1 (general endurance: 65–79% max HR or 1–2 mmol LA)	4	40–50
GE2 (general durance: 75–85% max HR or 2–4 mmol LA)	1	5–10
SP (special endurance: 85–95% max HR or 6–8 mmol LA)	2	5–10
Tempo (interval training: 85–95% max HR or 8–10 mmol LA)	1	3–5
SRE (strength running endurance: skips and hill running)	2	3
General fitness (supplementary session)	2	
Recovery (swimming, massage, cryotherapy)	1	
Day off (rest)	1	
Total training workouts per module	15	56–78

Abbreviation: LA—Lactate.

**Table 3:** Inspiratory muscle training program using PowerBreathe device.

Training Week	1	2	3	4	5	6	7	8
Training load	50% PI_max_	50% PI_max_	50% PI_max_	60% PI_max_	60% PI_max_	60% PI_max_	70% PI_max_	70% PI_max_
Training session (series × number of breaths)	2 × 30	2 × 30	2 × 30	2 × 30	2 × 30	2 × 30	2 × 30	2 × 30

**Table 4:** Inspiratory muscle training program using Threshold device.

Training Week	1	2	3	4	5	6	7	8
Training load	30% PI_max_	30% PI_max_	40% PI_max_	40% PI_max_	40% PI_max_	50% PI_max_	50% PI_max_	50% PI_max_
Training session (series × number of breaths)	2 **×** 30	2 **×** 30	2 **×** 30	2 **×** 30	2 **×** 30	2 **×** 30	2 **×** 30	2 **×** 30

**Table 5:** Inspiratory muscle training program for control group.

Training Week	1	2	3	4	5	6	7	8
Training load	15% PI_max_							
Training session (series × number of breaths)	1 × 60							

**Table 6:** Summary of measurements of physical performance parameters between tests (differences in results of test II to I and III to II).

Variable	PowerBreathe IMT Mean Change (Test II—Test I)		Threshold IMT Mean Change (Test II—Test I)		Control Group Mean Change (Test II—Test I)		PowerBreathe IMT Mean Change (Test III—Test II)		Threshold IMT Mean Change (Test III—Test II)		Control Group Mean Change (Test III—Test II)	
**sex**	W	M	W	M	W	M	W	M	W	M	W	M
**VO_2max_** **[mL/min]**	348.8 ** ^a^	294.4 *** ^c^	127.4 * ^b^	116.6 * ^b^	−26.7 ^a^	4 ^a^	21.5	0	−158.3 ** ^b^	−10	5.3	17.5^c^
**VO_2_/kg** **[(mL/mi)/kg]**	5.39 *** ^b^	4.08 * ^c^	1.45 ^b^	0.7	−0.5 ^a^	0.25 ^b^	1.21	−1.76 ^a^	1.85^c^	−1.34	−0.07	−0.05
**VCO_2max_ [mL/min]**	−157.5 ^a^	−70.2 ^a^	−176.0	11.06	70.0 ^a^	100.0	425.34 ** ^a^	−33.0	−51.0	−113.3 ^a^	6.0	205.5
**RER**	4.56 *** ^c^	3.97 ** ^b^	1.25 ^a^	4.37 *** ^c^	0.94 ^b^	0.11	2.26 ^b^	0.15	−1.29 ^a^	−0.64 ^a^	0.04	−0.38 ^a^
**V’E** **[L/min]**	14.67 *** ^c^	26.2 *** ^c^	11.25 *** ^c^	15.2 *** ^c^	0.33	0	0.66	4.2 ** ^a^	−11.3 *** ^c^	−12.4 *** ^c^	−1.0	−1.5 ^a^
**lactic acid [m/mL]**	−1.06 ** ^a^	−1.69 *** ^c^		−0.12	0.33	0.42 ^a^	0.03	0.04	0.38 ^b^	−0.14	−0.27	0.6 ^c^
**lactate threshold [min]**	2.94 ** ^c^	5.88 *** ^c^	0.7 ^b^	−0.41 ^a^	0.97 ^a^	−0.25 ^a^	0.25	1.35 ^a^	−0.52 ^a^	−1.24 ^c^	−0.13	−1.55 ^b^

Abb.: *—*p* ≤ 0.05, **—*p* ≤ 0.01, ***—*p* ≤ 0.001; ^a^ effect size > 0.2. ^b^ Effect size > 0.5, ^c^ effect size > 0.8. VO_2max_—maximal oxygen uptake, VCO_2max_—maximal carbon dioxide output, RER—respiratory exchange rate, V’E—minute ventilation.

**Table 7:** Summary of measurements of pulmonary parameters and respiratory muscle strength between tests (differences in results of test II to I and III to II).

Variable	PowerBreathe IMT Mean Change (Test II–Test I)		Threshold IMT Mean Change (Test II–Test I)		Control Group Mean Change (Test II–Test I)		PowerBreathe IMT Mean Change (Test III–Test II)		Threshold IMT Mean Change (Test III–Test II)		Control Group Mean Change (Test III–Test II)	
sex	W	M	W	M	W	M	W	M	W	M	W	M
VC [L]	0.38 * ^a^	0.15 ^a^	−0.09	0.55 *** ^c^	0.27 ^a^	0.18	0	0.05	−0.11 ^a^	0.02	−0.03	−0.08
FEV_1_ [L/s]	0.25 * ^a^	0.39 *** ^b^	0.05	0.04	−0.06	0.15	0.04	−0.15 ^a^	−0.11 ^a^	−0.22 ^a^	−0.03	−0.1
PEF [L/s]	0.19	0.2 a	0.04	−0.04	−0.08	−0.01	0.04	0.06	0.15 ^a^	0.26 ^a^	0.13	0.05
PI_max_ [kPa]	2.74 *** ^c^	2.31 *** ^c^	−0.72 ^b^	−0.28 ^c^	−0.43 ^a^	−0.15 ^a^	0.76 * ^a^	0.36	0.25	−0.3	−0.17	−0.3 ^b^
PE_max_ [kPa]	2.95 *** ^c^	2.95 *** ^c^	−1.2 * ^c^	0.56 ^a^	−0.17	0.05	0.19	0.4 ^a^	−0.12	−0.14	−0.36 ^a^	−0.2

Abb.: *—*p* ≤ 0.05, ***—*p* ≤ 0.001; ^a^ effect size > 0.2. ^b^ Effect size > 0.5, ^c^ effect size > 0.8. VC—vital capacity, FEV_1_—forced expiratory volume in one second, PEF—peak expiratory flow, PI_max_—maximum inspiratory pressure, PE_max_—maximum expiratory pressure.

## Data Availability

The data presented in this study are available on request from the corresponding author.

## Abbreviations

The following abbreviations are used in this manuscript:

VO_2max_	maximal oxygen consumption
VCO_2max_	carbon dioxide output
RER	respiratory exchange ratio
VE	minute ventilation
VC	vital capacity
FEV_1_	forced expiratory in one second
PEF	peak expiratory flow
PI_max_	inspiratory muscle strength
PE_max_	expiratory muscle strength
